# Chlorogenic acid improves the quality of boar semen processed in Percoll

**DOI:** 10.21451/1984-3143-AR2019-0021

**Published:** 2020-01-24

**Authors:** Stenia Severo Rabelo, Carla Oliveira Resende, Thais Preisser Pontelo, Bruna Resende Chaves, Bárbara Azevedo Pereira, William Eduardo da Silva, Juliano Vogas Peixoto, Luciano José Pereira, Márcio Gilberto Zangeronimo

**Affiliations:** 1 Departamento de Medicina Veterinária, Universidade Federal de Lavras, Lavras, MG, Brasil; 2 Departamento de Ciências da Saúde, Universidade Federal de Lavras, Lavras, MG, Brasil

**Keywords:** antioxidants, reproduction, sperm selection

## Abstract

This study aimed to evaluate if the addition of chlorogenic acid (ChA) to semen extenders improves the quality of cooled boar semen processed in Percoll. The experimental design was randomized blocks (ejaculates) in a 2×3 factorial (with or without Percoll, and three antioxidant systems: a negative control, without supplementation, a positive control – vitamin E, and ChA), totaling six treatments and 12 repetitions. ChA and vitamin E (VE) were added at 4.5 mg/ml and 400 μg/ml in extender, respectively. At 0, 48 and 72h of storage at 15ºC, 80 ml insemination doses each containing 2.0 billion sperm cells were submitted to centrifugation in Percoll. The use of Percoll impaired (P<0.01) all motility patterns but decreased (P<0.01) the number of abnormal cells at 0, 48 and 72h of storage. Both VE and ChA improved (P<0.05) the total motility after Percoll processing, but only in semen stored for 48h. The same effect was not observed (P>0.05) in semen stored for 72h. ChA improved (P<0.05) the total motility of the semen stored for 72h, but this effect was not observed (P>0.05) when the semen was processed in Percoll. The antioxidants had no effect (P>0.05) on the viability and integrity of the acrosome, but ChA reduced (P<0.05) the number of abnormal cells at 0h, while VE increased the number of abnormal cells in semen stored for 72h, independent of the use of Percoll. There was no effect (P>0.05) of antioxidants or Percoll on the concentration of malondialdehyde in seminal plasma. The use of Percoll had no effect (P>0.05) on the cholesterol efflux, but ChA increased (P<0.05) this parameter at 0h and reduced (P<0.05) in the semen stored for 72h not processed with Percoll. In conclusion, the addition of ChA to semen extenders improved the quality of boar semen processed or not in Percoll.

## Introduction

The need for higher quality boar semen destined for artificial insemination (AI) or *in vitro* fertilization (IVF) has led to the emergence of different methodologies of semen processing. One of these methods, the sperm selection technique using Percoll, has been widespread (Cesari et al., 2006; Zhou et al., 2010). This technique selects sperm subpopulations with increased physical integrity by their cell density. This procedure is suitable for obtaining cells with better core structure (Le Lannou and Blanchard, 1988) and for obtaining a smaller percentage of abnormal cells (Henkel and Schill, 2003).

However, negative effects on sperm motility have been reported (Guimarães et al., 2014), probably due to the formation of reactive oxygen species (ROS) during the centrifugation process (Iwasaki and Gagnon, 1992) or by the toxicity of Percoll (De Vos et al., 1997) which contains polyvinylpyrrolidone in its composition (Kato and Nagao, 2009). Besides participating in the sperm capacitation process, the presence of ROS could increase cellular abnormalities and acrosome changes, thereby limiting the quality of semen (Breininger et al., 2005). In this case, the addition of antioxidants in the extender could ease the sperm damage caused by ROS, improving the ability to maintain the quality and fertility of cooled semen for long time (Mendez et al., 2013; Pereira et al., 2014).

Vitamin E (Mendez et al., 2013) and chlorogenic acid (Basile et al., 2005; Pereira et al., 2014) are antioxidant substances that improve sperm quality when used during semen processing. Vitamin E (tocopherol) is a non-enzymatic antioxidant (Maneesh and Jayalekshmi, 2006) and is fat-soluble, which can protect the sperm from oxidative damage to DNA and the plasma membrane (Breininger et al., 2011). Mendez et al. (2013) reported that the addition of 400 µg/ml of vitamin E during the processing of boar insemination doses reduced the lipid peroxidation of sperm stored at 15 °C for 72 h. Furthermore, chlorogenic acid is a water soluble phenolic compound, that is widely distributed in nature and found primarily in vegetables (Basile et al., 2005). The presence of 4.5 mg/mL of chlorogenic acid in boar semen appears to improve the quality of insemination doses, especially when stored for periods longer than 24 h (Pereira et al., 2014).

Thus, this study aimed to determine whether the addition of vitamin E or chlorogenic acid extenders improves the quality of cooled boar semen processed with Percoll.

## Methods

### Semen collection and processing of insemination doses

The experiment was conducted at the Swine Reproduction Laboratory of the Veterinary Medicine Department of the Federal University of Lavras (UFLA) in Lavras, Minas Gerais, Brazil. All procedures were approved by the Ethics Committee on Animal Use of UFLA (protocol number 034/16).

A total of 16 ejaculates from four boars (four ejaculates each) of Duroc, Large White and Pietrain breeds, aged between 24 to 48 months and belonging to the Swine Experimental Center of UFLA, were used for this experiment. The animals were housed in individual grid stalls (2.75 m length x 2.10 m width and 1.30 m height) and fed daily with 3 kg of boar feed, divided into two feeding periods, at 8:00 am and at 2:00 pm. The water supply was provided *ad libitum*. Every animal was considered clinically healthy and of proven fertility. Weekly, three ejaculates were collected from each animal, always on alternate days.

Semen was collected by the gloved hand method in a specific room with the use of an immobile dummy. Before collection, the prepuce was cleaned and sanitized with paper towels. The semen was collected, and the gel fraction was discarded using a specific filter (Minitub do Brasil LTDA^®^, Porto Alegre, Brazil).

The experiment was conducted in a block design (ejaculates) in a 2×3 factorial scheme (with or without Percoll and three antioxidant systems: a negative control, without the addition of antioxidants; a positive control, with addition of VE; and chlorogenic acid), totaling six treatments and 12 repetitions of each ejaculate.

Before semen collection, three BTS (Beltsville Thawing Solution^®^ - Minitub of Brazil LTDA) extenders were prepared: without the addition of antioxidants, with 4.5 mg/ml of chlorogenic acid (chlorogenic acid crystalline, C3878 - *Sigma-Aldrich* ^®^) (Pereira et al., 2014) or with 400 µg/ml of vitamin E (DL-α-tocoferol acetate, T3251 - *Sigma-Aldrich* ^®^ *)* (Mendez et al., 2013), all maintained at 35 °C. Immediately after semen collection, sperm concentration was determined in a Neubauer chamber after diluting a sample of ejaculate in formaldehyde citrate solution at a ratio of 10:1000 (semen: formaldehyde citrate solution). Sperm count was estimated from the cell count in a 10-field Neubauer chamber under optical microscopy.

From each ejaculate, three inseminating doses of 40 mL containing two billion spermatozoa each were processed using one of the three extenders previously prepared. The insemination doses were then stored at room temperature and protected from light for 40 min, after which they were stored in a refrigerator at 15 °C.

At 0, 48 and 72 h of storage, 2.0 ml samples of semen were centrifuged (Heraeus Megafuge 16R Centrifuge, LED Thermo Electron GmbH, Osterode, Germany) at 1,000 × g for 15 minutes at 37 °C in 24 x 150 mm test tubes containing 2.0 ml of Percoll (Percolltm, GE Healthcare - Uppsala, Sweden) previously prepared in a 45/90 density gradient in 0.9% saline solution (Zhou et al., 2010). After this procedure, the samples were washed with 500 µL of BTS by centrifugation of 1,000 × g for 10 min at 37 °C. The sperm sediment at the bottom of the test tube was collected, diluted in 3.0 mL of BTS at 37 °C and maintained at this temperature for 15 min for microscopic and biochemical evaluations.

### Microscopic evaluations

All kinetic parameters were analyzed in the CASA system (Sperm Class Analyzer SCA 5.0, Microptic, Barcelona, Spain). For the test, 3.0 µL of semen was put in a specific glass plate (20 microns Leja^®^ - Microptic, Barcelona, Spain) preheated to 37 °C. The images were captured at 100 × magnification.

To assess the amount of abnormal cells, 200 µL semen samples were diluted into 700 µL of sodium-citrate aldehyde formalin solution at 3% (Pursel et al., 1972). Subsequently, 10 µL of this mixture was placed between a microscope slide and coverslip and evaluated under phase contrast microscopy (Olympus CX31, Olympus - Tokyo, Japan) at 1000 × magnification. Changes in the acrosome, head, middle piece and tail were recorded in a total of 200 cells. The values were expressed in percentages considering the total number of alterations detected in relation to the total number of cells analyzed.

Sperm viability was measured after mixing a drop of semen with a drop of eosin-nigrosin stain (Blom, 1950). After smearing on a microscope slide, a total of 200 cells was evaluated under a light microscope (Olympus CX31, Olympus - Tokyo, Japan) at 400 × magnification. Unstained cells were considered to have intact membranes and stained cells were considered dead. Values were expressed as the percentage of cells with intact membranes compared to the total number of cells counted.

To evaluate acrosome integrity, 10 µl of semen was added to 10 µl of dye POPE - Fast Green / Rose Bengal (Pope et al., 1991) and incubated for 60 s at 37 °C. After this period, 200 cells were then evaluated under phase contrast microscopy (Olympus CX31, Olympus - Tokyo, Japan) at 1000 × magnification. Cells with the acrosome region stained blue were considered to have intact acrosomes, and cells with the acrosome region unstained or only slightly stained were considered unhealthy.

### Biochemical evaluations

Biochemical analyses were performed on 1.0 ml samples of semen centrifuged at 3,000 × g for ten minutes (Ahluwalia and Holman, 1969). The supernatant was transferred to polypropylene tubes and frozen at -80 °C until the day of analysis.

To evaluate the concentration of malondialdehyde (MDA) in seminal plasma, the TBARS QuantiChromTM Kit Assay (DTBA- 100- Bioassay Systems - Hayward, USA) was used following the manufacturer's instructions (Brezezińska-Slebodzińska et al., 1995). For measurement of total cholesterol in the seminal plasma, the commercial kit Cholesterol Oxidase (Labtest Diagnosis S.A., Lagoa Santa, Brazil) was used following the manufacturer's instructions (Melo et al., 2009).

### Statistical analysis

The data were submitted to a normality test and homogeneity of variances by the Levene test, and then the analysis of variance (ANOVA). For the variables that did not meet the assumptions of ANOVA was used for the square root transformation of the data. The effect of males was considered in the model and all interactions were studied when P<0.05 by ANOVA. Means were compared by Tukey’s test at 5%. All statistical analyses were performed using the statistical package Action version 3.0.2.

## Results

**Figure 1:**
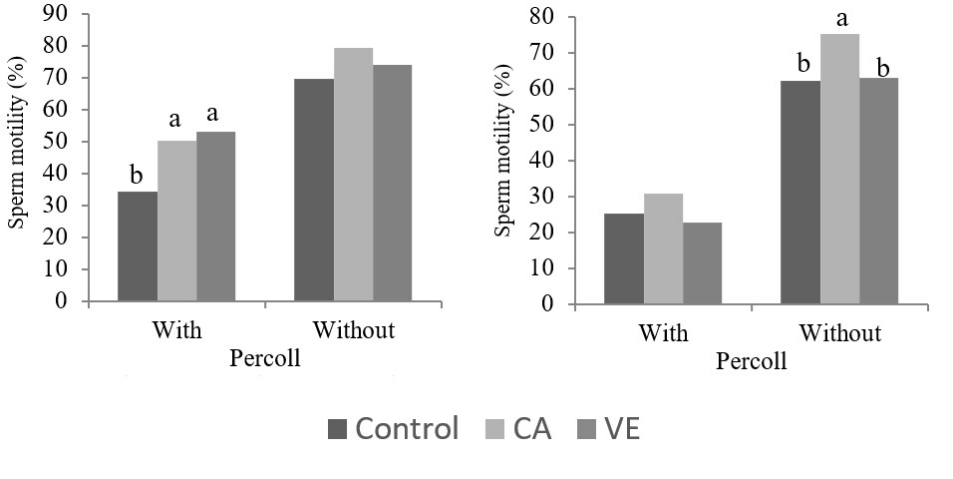
Total motility in the cooled boar semen after the addition of chlorogenic acid (CA) (4.5 mg/ml) or vitamin E (VE) (400 µg/ml) to the extender, with or without the use of the Percoll sperm selection technique (n=12). Means followed by different letters differ by Tukey’s test (P<0.05).

There was a significant interaction (P < 0.05) between the use of antioxidants and the Percoll technique regarding total motility in boar semen stored for 48 or 72 h (Table 1). Both vitamin E and chlorogenic acid improved (P < 0.05) the total motility when semen stored for 48 h was processed in Percoll. However, the same effect was not observed (P > 0.05) when the semen was stored for 72 h (Figure 1). With 72 h of storage, chlorogenic acid improved (P < 0.05) the total motility of semen not processed in Percoll; however, the same effect was not observed (P > 0.05) when Percoll was used.

**Table 1:** Motility patterns of cooled boar semen after the addition of chlorogenic acid (CA) (4.5 mg/ml) or vitamin E (VE) (400 µg/ml) to the extender, with or without the use of the Percoll sperm selection technique (n=12).

Storage time	Percoll (Pe)			Antioxidant (Ao)			CV (%)	P value		
	**With**	**Without**		**Without**	**CA**	**VE**		**Pe**	**Ao**	Pe*Ao
	Total motility (%)									
0 hours	84.1	88.1		83.8	88.0	86.8	8.96	0.04	0.16	0.98
48 hours	46.0	74.3		53.6^b^	65.4^a^	64.1^a^	10.72	<0.01	<0.01	0.03
72 hours	26.3	66.8		44.8^b^	54.2^a^	43.9^b^	10.00	<0.01	<0.01	0.06
	Progressive motility (%)									
0 hours	30.8	46.4		41.9	46.7	43.9	14.74	<0.01	0.14	0.21
48 hours	13.2	27.6		22.4	24.6	19.9	16.95	<0.01	0.28	0.93
72 hours	1.82	12.6		8.69	8.79	9.82	11.04	<0.01	0.30	0.12
	VAP - Velocity average path (µm/s)									
0 hours	39.5	47.5		42.9	44.6	43.6	9.64	<0.01	0.77	0.87
48 hours	21.9	31.9		28.0	25.0	28.8	15.97	<0.01	0.12	0.14
72 hours	12.5	26.0		19.9	17.8	21.1	12.49	<0.01	0.14	0.16
ALH - amplitude of lateral head displacement (µm)										
0 hours	2.01	2.28		2.22	2.10	2.13	10.09	<0.01	0.69	0.64
48 hours	1.51	1.65		1.61	1.52	1.63	7.48	0.01	0.30	0.36
72 hours	1.28	1.61		1.55	1.38	1.43	12.12	<0.01	0.12	0.13
	LIN - Linearity (%)									
0 hours	0.524	0.549		0.527	0.569	0.517	11.32	0.10	0.12	0.19
48 hours	0.362	0.539		0.436	0.485	0.444	14.15	<0.01	0.49	0.18
72 hours	0.353	0.511		0.401	0.455	0.451	11.28	<0.01	0.25	0.21
	WOB - Wobble (%)									
0 hours	0.711	0.750		0.730	0.745	0.720	5.85	<0.01	0.17	0.23
48 hours	0.553	0.714		0.626	0.650	0.638	8.95	<0.01	0.86	0.11
72 hours	0.511	0.693		0.578	0.610	0.633	7.15	<0.01	0.12	0.21

Means followed by different letters in the line differ by Tukey’s test (P<0.05). CV: Coefficient of variance.

No interaction (P > 0.05) between antioxidants and the Percoll technique was detected for other motility patterns, sperm viability, acrosomal integrity, percentage of abnormal cells (Table 2) or MDA concentration in the seminal plasma (Table 3). The Percoll technique was efficient (P < 0.01) in reducing the percentage of abnormal cells, but damaged (P < 0.01) all motility patterns. The viability was improved (P < 0.01) by the Percoll technique only in the semen stored for 48 h. There was no effect (P > 0.05) of the Percoll technique on the MDA concentration in the seminal plasma.

**Table 2:** Sperm viability, acrosome integrity and percentage of abnormal cells in the cooled boar semen after the addition of chlorogenic acid (CA) (4.5 mg/ml) or vitamin E (VE) (400 µg/ml) to the extender, with or without the use of the Percoll sperm selection technique (n=12).

Storage time	Percoll (Pe)			Antioxidant (Ao)			CV (%)	P value		
	**With**	**Without**		**Without**	**CA**	**VE**		**Pe**	**Ao**	Pe*Ao
	Sperm viability (%)									
0 hours	87.4	86.4		86.8	87.1	86.8	4.73	0.28	0.95	0.17
48 hours	86.4	82.1		83.4	84.0	85.4	5.51	<0.01	0.31	0.26
72 hours	83.8	81.9		83.9	81.3	83.3	5.01	0.09	0.14	0.50
	Acrosome integrity (%)									
0 hours	94.9	96.6		95.7	95.7	95.9	1.60	<0.01	0.86	0.31
48 hours	91.0	90.8		90.3	91.7	90.7	3.93	0.80	0.37	0.29
72 hours	89.7	89.9		89.3	89.9	90.3	3.40	0.76	0.62	0.58
	Abnormal cells (%)									
0 hours	4.12	6.44		5.07^ab^	3.54^b^	7.24^a^	9.07	<0.01	<0.01	0.12
48 hours	5.81	10.00		8.41	7.81	7.50	8.74	<0.01	0.79	0.42
72 hours	7.25	10.12		7.43^b^	6.11^b^	12.51^a^	7.81	<0.01	<0.01	0.15

Means followed by different letters in the line differ by Tukey’s test (P<0.05). CV: Coefficient of variance.

**Table 3:** Malondialdehyde level (µM) in cooled boar semen after the addition of chlorogenic acid (CA) (4.5 mg/ml) or vitamin E (VE) (400 µg/ml) to the extender, with or without the use of the Percoll sperm selection technique (n=12).

**Storage time**	**Percoll (Pe)**			**Antioxidant (Ao)**			**CV (%)**	**P value**		
	**With**	**Without**		**Without**	**CA**	**VE**		**Pe**	**Ao**	Pe*Ao
0 hours	0.910	0.864		0.884	0.868	0.907	15.57	0.57	0.98	0.24
48 hours	0.716	0.858		0.767	0.813	0.775	22.42	0.10	0.84	0.88
72 hours	0.708	0.809		0.701	0.866	0.726	15.46	0.13	0.13	0.58

No difference by the F-test (P>0.05).

The antioxidants had no effect (P > 0.05) on sperm viability or acrosome integrity. Independent of the use of the Percoll technique, chlorogenic acid reduced (P < 0.05) the percentage of abnormal cells in fresh semen (0 h), while vitamin E increased (P < 0.05) this parameter in the semen stored for 72 h. There was no effect (P < 0.05) of antioxidants on the MDA concentration in seminal plasma.

A significant interaction (P < 0.05) between antioxidants and the Percoll technique was observed for the cholesterol concentration in seminal plasma. In fresh semen, chlorogenic acid increased (P < 0.05) the cholesterol levels in seminal plasma only when the Percoll technique was used. For semen already stored for 72 h, when compared to the control (without antioxidant addition), chlorogenic acid reduced (P < 0.05) the concentration of cholesterol only in semen not processed in Percoll. The Percoll technique had no effect (P > 0.05) on the cholesterol efflux of boar semen.

## Discussion

While the Percoll technique was efficient in selecting sperm cells with fewer abnormalities, it reduced all sperm motility patterns regardless of the addition of antioxidants to the extender. This result indicates that both chlorogenic acid and vitamin E were not effective in mitigating the negative effects caused by Percoll.

The Percoll technique is recommended for IVF (Matás et al., 2011). In these cases, the physical cellular integrity is more important than the locomotor ability of the spermatozoa. However, use of the Percoll technique to produce insemination doses for *in vivo* fertilization is not yet feasible.

Several studies have shown that the use of substances with antioxidant properties in the extender improves the quality of thawed boar semen (Ma et al., 2015; Shen et al., 2016), whose manipulation is intense. However, there has not been enough information to demonstrate these findings in semen processed in Percoll. The benefits expected from the use of antioxidants added to the extender could extend to the use of Percoll to produce semen for AI programs.

According to Iwasaki and Gagnon (1992), the manipulation of semen can result in the formation of ROS. The same also occurs with cooled boar semen (Martín-Hidalgo et al., 2013). It is known that ROS can damage the sperm membrane and, thus, reduce sperm motility and increase the number of damaged cells (De Lamirande and Gagnon, 1992). ROS also induce irreversible changes in the proteins and nucleic acids of spermatozoa, stimulating apoptosis and cell death (Lewis and Aitken, 2005).

However, an increased MDA concentration in seminal plasma due to the processing of semen in Percoll could not be verified in the present study. Thus, it is believed that the adverse effects of this technique on semen quality are more related to the toxicity of polyvinylpyrrolidone present in Percoll (Barak et al., 2001), although there is evidence that MDA can react with proteins and amino acids present in the media (Chio and Tappel, 1969). In this case, the use of MDA identification techniques was not sufficient to evaluate the presence of ROS in the media. Moreover, the reaction between MDA and proteins is very complex and is dependent on temperature, pH and the incubation period of samples (Shin et al., 1972). However, the ability of MDA to react with proteins in the media has not been identified in boar semen. Thus, the results of this study suggest that the toxic effects of polyvinylpyrrolidone may have more strongly influenced the semen quality.

Aside from the use of the Percoll technique, both chlorogenic acid and vitamin E have shown positive effects on boar semen quality until 48h of storage. Both substances have been studied as protective agents of oxidative stress in semen, probably due to their inhibitory effects on ROS (Mendez et al., 2013; Pereira et al., 2014). Until 72h, only ChA has a positive effect. Pereira et al. (2018) have shown the benefits of ChA until 72h of storage of cooled boar semen, suggesting the potential use of ChA as a protective agent to a greater degree than vitamin E, especially for storage times close to 72h or more. In fact, numerically there were fewer abnormal cells in semen stored for 72h when compared to semen without addition of antioxidants. The increase in the number of abnormal cells with vitamin E in semen stored for 72 h was also observed. Mendez et al. (2013) observed a linear increase in acrosoma damage by increasing the concentration of this vitamin from 100 to 400 µg/mL. The authors attribute this result to the fact that high concentrations of this vitamin may have been oxidized in samples stored for a long time, which could have changed the characteristics of the plasma membrane, affecting its fluidity and, consequently, its cellular structure (Dalvit et al., 1998). In this case, studies should be conducted to prove any long-term toxic effects of this vitamin.

**Figure 2:**
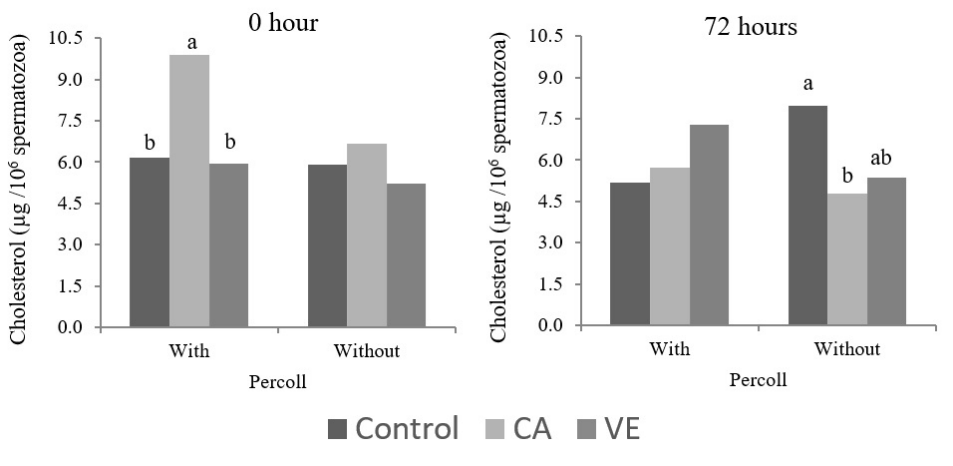
Cholesterol concentration in seminal plasma of cooled boar semen after the addition of chlorogenic acid (CA) (4.5 mg/ml) or vitamin E (VE) (400 µg/ml) to the extender, with or without the use of the Percoll sperm selection technique. Means followed by different letters differ by Tukey’s test (P<0.05).

Vitamin E has already been widely studied because of its protective effects against oxidative damage in sperm (Jeong et al., 2009; Satorre et al., 2012). Similarly, chlorogenic acid has recently been proven to benefit cellular functionality and animal health (Darvesh et al., 2010; Sato et al., 2011) and also the quality of boar semen (Pereira et al., 2014), probably due to its antioxidant properties that reduce ROS. It is known that ROS are able to induce sperm capacitation by mechanisms not fully elucidated (O’Flaherty et al., 2006). In this sense, one of the events that occurs during sperm capacitation is the membrane cholesterol output (Signorelli et al., 2012). In the present study, it was found that the Percoll technique was not sufficient to induce sperm capacitation (Figure 2), except in fresh semen (0 h) when chlorogenic acid was added to the extender. This result suggests that the presence of chlorogenic acid in semen coupled with the stress caused by the Percoll technique enables some mechanism involved in sperm cholesterol efflux.

On the other hand, this effect of chlorogenic acid was not observed in semen stored for 72 h. Otherwise, when compared to the control group (without addition of antioxidants), chlorogenic acid reduced the cholesterol efflux, but only in semen not processed in Percoll. The same was not observed with vitamin E. This result suggests a late antioxidant effect of chlorogenic acid when compared to vitamin E, mitigating the oxidative stress that occurs in semen stored for prolonged periods. In fact, chlorogenic acid resulted in increased motility only in the semen stored for 72 h and not processed in Percoll (Figure 1). This chlorogenic acid effect was also observed by Pereira et al. (2014). However, the benefits provided by chlorogenic acid to the cooled boar semen were not enough to prevent the loss of sperm quality after processing in Percoll.

In general, the results of the present study showed that the greatest benefit of the Percoll selection method was to reduce the percentage of abnormal sperm cells and that the addition of antioxidants to the semen extender can mitigate the harmful effects caused by the use of this technique. However, these benefits were not sufficient enough to prevent the loss of sperm quality after using Percoll. In this sense, further studies should be conducted in an attempt to improve the quality of boar semen processed in Percoll for later use in AI programs.

## Conclusion

The addition of chlorogenic acid to the semen extender improves the quality of boar semen processed or not in Percoll sperm selection technique, better than those obtained with vitamin E supplementation. However, the benefits are not enough to prevent the loss of sperm quality after using this technique.
